# Strategies for Fostering HPV Vaccine Acceptance

**DOI:** 10.1155/IDOG/2006/36797

**Published:** 2006

**Authors:** Bernard Gonik

**Affiliations:** Department of Obstetrics and Gynecology, School of Medicine, Wayne State University, Detroit, MI 49201, USA

## Abstract

Vaccines that protect against infection with the types of human papillomavirus (HPV) commonly associated with cervical cancer (HPV 16 and 18) and genital warts (HPV 6 and 11) are expected to become available in the near future. Because HPV vaccines are prophylactic, they must be administered prior to exposure to the virus, ideally during preadolescence or adolescence. The young age of the target vaccination population means that physicians, parents, and patients will all be involved in the decision-making process. Research has shown that parents and patients are more likely to accept a vaccine if it is efficacious, safe, reasonably priced, and recommended by a physician. Widespread education of physicians, patients, and parents about the risks and consequences of HPV infection and the benefits of vaccination will be instrumental for fostering vaccine acceptance.

## INTRODUCTION

Human papillomavirus (HPV) is the most common sexually transmitted infection (STI) and a known risk factor for cervical cancer [1, 2]. HPV is highly prevalent in younger populations, with prevalence rates of approximately 50% in sexually active adolescent girls and young women [3]. Early age of first sexual intercourse is associated with greater susceptibility to HPV infection, possibly due to cervical immaturity [4]. Current estimates suggest that approximately 15% of sexually active adults in the United States have clinical or subclinical infections [5]. Despite the fact that most sexually active individuals will become exposed throughout their lives, knowledge about HPV and the diseases that HPV causes is generally very limited [6].

The average age of first sexual intercourse inNorth America is approximately 16 years for both men and women [7]. To provide the greatest public health benefit, HPV vaccines must be administered prior to initiating sexual activity and hence would be most effective if offered during early adolescence. Because gynecologists often serve as the sole health care provider for many women, they are in an excellent position to disseminate information about the risks and consequences of HPV infection as well as provide information about HPV vaccines that will soon become commercially available.

## GENERAL AWARENESS OF HPV

Population awareness of the risks associated with acquiring HPV and the consequences associated with infection is low.

Numerous studies have demonstrated that most patients are unaware of HPV and its association with genital warts and cervical cancer [2, 6, 8–14]. Furthermore, awareness has not improved in the past decade: approximately 33% of women and half of men surveyed have never heard of HPV [9, 11]. Nonetheless, there is a desire for more information about HPV [15]. Women want to know how HPV is transmitted, including whether intercourse is required to transmit disease, and how they and their sexual partners can prevent becoming infected. Additional information of interest includes whether condoms protect against the virus, how HPV is detected, the likelihood of spontaneous resolution of infection, and the risk of cervical cancer [16].

## HPV VACCINE ACCEPTANCE

Vaccines designed to reduce the incidence of HPV infection are in late stages of clinical development and studies to date have demonstrated that these vaccines are safe and highly efficacious. Widespread acceptance of HPV vaccines are likely to lend enormous health benefits by decreasing morbidity and mortality associated with cervical cancer and by reducing the psychosocial burden of both genital warts and abnormal Papanicolaou (Pap) test results. Savings in health care expenditures, including treatments for genital warts, preinvasive cervical lesions, and cervical cancer would also be considerable [17].

Historically, however, vaccine availability has not always translated to widespread use. Underestimates of both the level of risk and the severity of HPV-associated disease may become barriers to vaccine acceptance [18]. Hence, physician, parent, and patient education about HPV and HPV vaccines will be crucial to effectively implement HPV vaccination programs.

### Vaccine acceptability among individuals

Attitudes regarding HPV vaccine acceptance can be assessed based on acceptability of previous vaccines and through questionnaires completed by patients, parents, and health care providers. For example, Kahn et al presented questionnaires to 52 young women regarding HPV vaccination, and found that most viewed vaccination positively [19]. Furthermore, most reported a high intention of receiving the vaccine, both for themselves and their hypothetical daughters. Knowledge about HPV and the vaccine, personal beliefs about vaccination, beliefs that others would approve of vaccination, higher number of sexual partners, and perceived support of provider, partner, and parents were all significantly associated with intention to receive the vaccine [19]. In a separate study of 256 college students, 74% endorsed HPV vaccination [20]. Of those surveyed, acceptance significantly correlated with higher number of sexual partners, parental support, endorsement of universal HPV vaccination, low cost, and vaccine safety. Hoover et al evaluated knowledge of HPV and attitudes toward HPV vaccination and clinical trial participation among 60 female adolescents and young adults [21]. Almost all of the participants expressed interest in receiving a vaccine that would prevent cervical cancer and genital warts. Another study examined the attitudes about hypothetical HPV vaccines in adolescent (*n* = 20) and adult women (*n* = 20) attending two urban STI clinics [22]. Here, the idea of an HPV vaccine was favorably received and several factors affected potential acceptance, including vaccine efficacy, physician endorsement, and cost.

### Vaccine acceptability among parents

Vaccine acceptance among parents is not universal. Although, in general, overall confidence in vaccines remains high, many Americans distrust public health policy and refuse vaccination on these grounds [23]. Concern over potential side-effects from vaccines is a common barrier to vaccination [24]. Parents may also object to vaccination for religious or philosophical reasons [25].

To overcome these barriers, clinicians should informparents of the prevalence of HPV among adolescents and discuss the potential consequences of forgoing vaccination and becoming infected. Although many childhood vaccines are for diseases that are now rare, HPV is highly prevalent and educational efforts should stress that most sexually active adolescents will acquire HPV.

The sexual nature of HPV infections may introduce unique barriers to parental consent not previously encountered with other vaccines. Parents may feel that consenting to a vaccine for an STI may inadvertently encourage their adolescent children to engage in sexual intercourse. Similarly, parentsmay feel that vaccination at an early age will encourage earlier sexual debut [26]. Acceptance of the vaccine represents an acknowledgment of risk of infection [27], and some parents may have difficulty accepting the fact that their children are approaching an age at which sexual activity is often initiated.

Contrary to these beliefs, several studies have suggested that the sexually transmitted nature of HPV may not pose a major obstacle toHPV vaccine acceptance. In one study, 70% of parents approved of vaccination for STIs [18, 28]. Desire to protect their children, concern about specific disease characteristics, and personal experience with an STI were directly correlated with vaccine acceptance. Rejection was associated with the perception that their child was at low risk for infection or with the parent having a low concern about severity of the disease. In a similar study, Zimet et al questioned 278 parents about their attitudes toward adolescent vaccination, incorporating nine hypothetical STI scenarios defined along four different dimensions: mode of transmission (STI vs non-STI), severity (curable/chronic/fatal), vaccine efficacy (50%, 70%, 90%), and behavioral method for prevention (available/not available) [27]. Interestingly, whether a disease was sexually transmitted did not affect the parents' decision. Instead, severity of the hypothetical disease and vaccine efficacy predicted vaccine acceptance [27].

When parents who were undecided about HPV vaccination were provided with a basic information sheet about HPV and HPV vaccines, they became significantly more likely to support HPV vaccination [26]. Physician endorsement and school requirements have also been identified as important catalysts for parental vaccine acceptance [29]. Conversely, information acquired from friends, relatives, or advertisements had a marginal impact.

### Vaccine acceptability among health care professionals

Endorsement by professional organizations is a major predictor of HPV vaccine acceptance. One study concluded that a recommendation from the American College of Obstetricians and Gynecologists would be the most important factor influencing whether gynecologists would recommend vaccination [29]. If the endorsements included a specific age for vaccination, this would undoubtedly increase acceptance of early-adolescent HPV vaccination.

**Figure 1:**
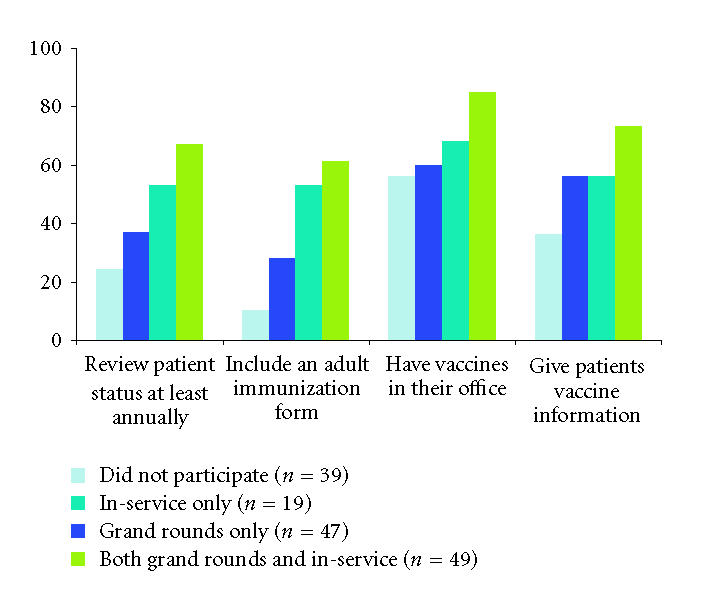
Interventions, participation, and office practice. Gynecologists who attended in-service or grand rounds educational lectures were more likely to vaccinate or provide vaccine information to their patients than those who did not [30].

Educational initiatives targeting health care professionals have demonstrated effectiveness in fostering vaccine acceptance. A study to measure the effectiveness of a statewide peer educational program on changes in clinician immunization practice patterns and behaviors was conducted between June 1999 and October 2000 (Figure 1) [30]. Interventions included hospital grand rounds lectures and officebased small group in-service sessions. Forty-two percent of the 532 providers who attended the 16 grand rounds lectures completed both the pre- and posttests; the response rate was the same for the 368 physicians who attended the 15 office-based sessions. Knowledge (*P* < .05) and practice attitudes (*P* < .01) pertaining to vaccine-preventable disease and immunization improved after attending a lecture or in-service session. Grand rounds were more effective than in-service sessions at convincing physicians to change their vaccine-preventable disease office practices (*P* < .01), but everyone who participated in one or more interventions had better knowledge recall, improved attitudes toward vaccinepreventable disease, and practice patterns that were more likely to improve patient vaccine awareness compared with those who did not participate (*P* < .05). Thus, educational interventions can positively influence immunization-related practice patterns.

Educational efforts aimed at health care professionals should include physician assistants and nurse practitioners, as well as physicians. Educating patients about HPV and the HPV vaccine will be time consuming, and the assistance these groups can provide will prove to be invaluable.

Clinicians should provide information in an individualized fashion, tailoring educational sessions according to the patient's background, age, and literacy level [16]. An effort should be made to provide needed information to parents and adolescents without creating needless anxiety over the situation [31]. Nonetheless, it is crucial that the high risk of infection, the frequent negative consequences associated with infection, and the importance of vaccinating in early adolescence, before the individual has become sexually active, be made clear to the parent.

## CONCLUSIONS

Prophylactic HPV vaccines may soon become available for public distribution; however, general knowledge about HPV and HPV-associated disease is limited and may affect vaccine acceptability. Research suggests that vaccine acceptance will be maximized by effectively communicating the risks associated with HPV infection and the benefits of vaccination. Educational initiatives targeted towards patients, parents, and health care providers will play key roles in fostering positive attitudes towards vaccination.
